# Cortical and Clonal Contribution of Tbr2 Expressing Progenitors in the Developing Mouse Brain

**DOI:** 10.1093/cercor/bhu125

**Published:** 2014-06-13

**Authors:** Navneet A. Vasistha, Fernando García-Moreno, Siddharth Arora, Amanda F.P. Cheung, Sebastian J. Arnold, Elizabeth J. Robertson, Zoltán Molnár

**Affiliations:** 1 Department of Physiology, Anatomy and Genetics, University of Oxford, Oxford, UK; 2 Oxford Centre for Industrial and Applied Mathematics (OCIAM), University of Oxford, 24-29 St Giles’, Oxford, UK; 3 Renal Department, University Medical Centre, Centre for Clinical Research, Breisacher Strasse 66, 79106 Freiburg, Germany; 4 BIOSS Centre of Biological Signalling Studies, Albert-Ludwigs-University, Freiburg, Germany; 5 Sir William Dunn School of Pathology, Oxford, UK

**Keywords:** Cerebral cortex, clonal analysis, *CLoNe*, fate-mapping, intermediate progenitors, subventricular zone

## Abstract

The individual contribution of different progenitor subtypes towards the mature rodent cerebral cortex is not fully understood. Intermediate progenitor cells (IPCs) are key to understanding the regulation of neuronal number during cortical development and evolution, yet their exact contribution is much debated. Intermediate progenitors in the cortical subventricular zone are defined by expression of T-box brain-2 (Tbr2). In this study we demonstrate by using the *Tbr2^Cre^* mouse line and state-of-the-art cell lineage labeling techniques, that IPC derived cells contribute substantial proportions 67.5% of glutamatergic but not GABAergic or astrocytic cells to all cortical layers including the earliest generated subplate zone. We also describe the laminar dispersion of clonally derived cells from IPCs using a recently described clonal analysis tool (*CLoNe*) and show that pair-generated cells in different layers cluster closer (142.1 ± 76.8 μm) than unrelated cells (294.9 ± 105.4 μm). The clonal dispersion from individual Tbr2 positive intermediate progenitors contributes to increasing the cortical surface. Our study also describes extracortical contributions from Tbr2+ progenitors to the lateral olfactory tract and ventromedial hypothalamic nucleus.

## Introduction

Intermediate progenitor cells (IPCs) are transient transit-amplifying progenitors present during neocortical neurogenesis that arise by asymmetric divisions of radial glial progenitors (Haubensak et al. 2004; Miyata et al. 2004; Noctor et al. 2004). These progenitors reside in the subventricular zone (SVZ) where the majority terminally divide to produce a pair of neurons (Noctor et al. 2004). Additionally, they differ from radial glial progenitors in lacking apico-basal polarity and largely undergoing terminal divisions (Noctor et al. 2004).

Intermediate progenitors are defined by expression of the long noncoding RNA Svet1 (Tarabykin et al. 2001) and by transcription factors such as *T-box brain-2* (*Tbr2*) (Englund et al. 2005) and *Cux2* (Zimmer et al. 2004). Both of these transcription factors are known to play significant role in IPC biology and mice deficient for *Tbr2* and *Cux2* display defects in IPC specification and subsequent generation of neurons (Arnold et al. 2008; Cubelos et al. 2008; Sessa et al. 2008).

Evolutionarily, IPCs are thought to be instrumental in the expansion of the neocortical thickness by amplifying the neuronal output from radial glia (Martínez-Cerdeño et al. 2006; Cheung et al. 2010). It has been shown that nonventricular surface divisions increase at late embryonic stages, during the formation of upper-layers (Martínez-Cerdeño et al. 2006), therefore relating IPC-derived neurons to supragranular layers. Previous fate-mapping using a *Neurod6* (*NEX*) driven Cre mouse line has shown IPC contribution towards the formation of upper layers (Wu et al. 2005). This is further supported by the large decrease in the upper cortical layer thickness and Cux1+ cells in *Tbr2* conditionally deleted mice (Arnold et al. 2008). In macaque fetal cortices, during periods of infragranular layer generation, nearly 30% of tritiated thymidine is incorporated into nonventricular zone progenitors (inner/outer SVZ) (Lukaszewicz et al. 2005). With recent studies showing large numbers of IPCs in both these regions (Hansen et al. 2010; Reillo et al. 2010; Fietz and Huttner 2011; García-Moreno et al. 2012), it suggests that IPCs could also be involved in formation of infragranular layers, at least in larger brains.

In mice, using a *Tbr2::GFP* (BAC) transgenic line, it has been shown that a significant Tbr2+ population exists at E10–E11 suggesting that IPCs might also contribute towards genesis of infragranual layers (Kowalczyk et al. 2009). Since impermanent labeling in *Tbr2::GFP* brains precludes studying the final laminar positions of the cells derived early from Tbr2+ IPCs, the ultimate fate, distribution and proportions of all Tbr2+ progenitors currently remains elusive.

A quantitative understanding of the neuronal output from IPCs towards cortical neurogenesis is thus at present absent and impeding our understanding of neuronal cell number regulation during development and evolution of the forebrain. Moreover, no previous study has examined the clonal generation of neurons from individual Tbr2+ IPCs. Further, while neural progenitors across species share some characteristics, their diversity in primate cortices has only recently been described (Betizeau et al. 2013; Cunningham et al. 2013). A quantitative comparison of rodent and primate progenitors will hence lead to insights into the evolution of the neocortex.

We thus investigate IPC dependent lineages using the *Tbr2^Cre^* and *Ai9-tdTomato* mouse lines (Madisen et al. 2009; Costello et al. 2011). In addition, we use a novel clonal analysis method: *CLoNe* that is based on stable integration of Cre-inducible fluorophores by transposases using in utero electroporation (García-Moreno et al. 2014) to clonally follow IPC contribution towards the cortical layers.

## Materials and Methods

### Mouse Strains

All animal experiments were conducted in accordance with the UK Animals (Scientific Procedures) Act (1986). C57BL6/J background mice were used throughout the study. The date of vaginal plug was observed at E0.5 and embryos accordingly timed.

*Tbr2^Cre^* mice were bred with *Ai9-tdTomato* reporter mice to label all neurons derived from the Tbr2+ IPCs. Pups born from this mating were perfused at P7 (*N* = 3) and P21 (*N* = 4) and mice were used for cell counts and immunostaining.

For layer-wise comparison in the *Tbr2^flox/flox^*, *Sox1^Cre^* mice were crossed with *Tbr2^flox/flox^* (Arnold et al. 2008) and animals with deletions in both alleles were selected. Pups (*N* = 3) were perfused at P8.

Images were taken at the level of the S1 cortex.

### Fluorescent In Situ Hybridization (FISH)

Probes were prepared by ligating into pGEMT (Promega), the PCR product from the following primers on a known Cre expressing plasmid.

Forward (5′-3′): GCCGCCACCATGGCCAATTT

Reverse (5′-3′): GCGGCCGCTATCACAGATCT

Standard ISH protocols were used as described in Hoerder-Suabedissen et al. (2009). Briefly, DIG-labeled riboprobes were synthesized using T7 and SP6 RNA polymerase according to manufacturer’s protocols (Roche). Tissue was fixed with 4% paraformaldehyde (PFA) (Sigma Aldrich) for 4 h, cryoprotected with 30% sucrose overnight and frozen in OCT (TissueTek). Sections of 16 μm thickness were cut on a cryostat (Jung CM3000; Leica) and stored at −80° until use.

Sections were postfixed with 4% PFA for 5 min, deproteinized with 0.1 N HCl for 5 min and acetylated with acetic anhydride (0.25% in 0.1 M triethanolamine hydrochloride). Prehybridization was carried out at room temperature (RT) for at least 1 h in a solution of 50% formamide, 200 μg/mL *E. coli* transfer RNA, 1× Denhardt's solution, 10% dextran sulfate, 10 mM Tris (pH 7.6), 600 mM NaCl, 0.25% sodium dodecyl sulfate, and 1 mM ethylenediaminetetra-acetic acid (EDTA). Hybridizations were carried out overnight at 65°C with riboprobes diluted in the prehybridization solution. Sections were washed in 30 mM NaCl/3 mM sodium citrate first at 65°C for an hour and then at RT for 5 min. Slides were blocked with 1% blocking reagent (Roche) in 100 mM Tris–Cl (pH 7.5) and 150 mM NaCl. This was followed with overnight incubation in anti-DIG antibody (1:500, Horseradish peroxidase conjugated, Roche) at 4°C. Sections were developed using the TSA-plus Cyanine 3 Kit (PerkinElmer) according to the manufacturer’s instructions, counterstained with 4′,6-diamidino-2- phenylindole (DAPI) and mounted with phosphate-buffered saline (PBS).

### Plasmids

Plasmids used for clonal analysis have been described previously (García-Moreno et al. 2014) The transposase expressing vector (or mPB), was kindly provided by Wellcome Trust Sanger Institute (Yusa et al. 2009) and is required for insertion of the fluorophore plasmids into the genome of progenitor cells.

### In Utero Electroporation

In-utero electroporation was performed as reported previously (García-Moreno et al. 2014) with modifications. Time-mated pregnant dames were administered Vetergesic intraperitoneally (0.08 mg/kg) for analgesia prior to anesthesia with Isoflurane. A midline laparotomy was undertaken and uterine horns exposed gently. DNA (mixed with Fast Green, Sigma) was injected into the lateral ventricles using pulled glass capillaries (Harvard Apparatus). Square pulses (at 30–35 V, 5 × 50 ms with 950 ms resting periods) were delivered using tweezer electrodes (CUY650P3, Nepagene) connected to a square pulse generator (Electro Square Porator, Harvard Apparatus). Warm PBS was used to keep the embryo sacs at near body temperature along with a warm blanket.

Following electroporation, the uterine horns were gently placed back in the abdominal cavity. Surgical incisions were sutured (Vicryl 4/0, Ethicon) and the skin held together using surgical clips. Animals were allowed to recover after removal of anesthesia and kept on heating pads until they regained movement.

### Immunohistochemistry

Drop-fixation of embryonic brains was carried out at 4°C overnight in 4% paraformaldehyde (PFA; Sigma-Aldrich) and subsequently stored in PBS with 0.05% sodium azide (Sigma). For postnatal ages, animals were transcardially perfused first with cold PBS and then with cold 4% PFA before collecting the brains. Brains were sectioned on the Leica VT1000S vibrating microtome at 40–50 μm and collected in PBS. Sections were washed in PBS thrice for 5 min each and permeabilised in PBS–Triton-X (0.1%; PBST) for 30 min. Following blocking with 5% serum (in PBS-T) for 2 h, primary antibody diluted in PBS-T with 1% serum was added and the sections incubated overnight at 4°C. The following day, the sections were again washed before incubation with secondary fluorescent-labeled antibodies (Molecular Probes, Invitrogen) for 2 h at room temperature. Sections were counter-stained with DAPI (Invitrogen) and mounted on slides with PBS and covered with a coverslip. The edges were sealed with clear nail-varnish and allowed to dry.

### List of Antibodies Used for Immunohistochemistry

**Table:** 

Antibody	Source	Concentration
	Primary antibodies	
Chick anti-EYFP	GFP-1020; Aves Lab	1/1000
Chick anti-Tbr2	AB15894; Millipore	1/250
Goat anti-Nurr1	RD Systems	1/200
Mouse anti-Calbindin	300; Swant D-28k	1/500
Mouse anti-Otx1		1/1000
Mouse anti-Satb2	ab51502; Abcam	1/200
Mouse anti-DsRed (tdTomato)	632393; Clontech	1/500
Rabbit anti-ChAT	AB-N34; ATS	1/2000
Goat anti-CTGF	Sc-14939; Santa Cruz	1/1000
Rabbit anti-Cux1	sc-13024; Santa Cruz	1/200
Rabbit anti-GABA	A2052; Sigma	1/1000
Rabbit anti-GFAP	G9269; Sigma	1/500
Rabbit anti-Ki67	ab15580; Abcam	1/500
Rabbit anti-Tbr1	ab31940; Abcam	1/1000
Rat anti-Ctip2	ab18465; Abcam	1/500
Sheep anti-TH	ab113; Abcam	1/1000
	Secondary antibodies	
Donkey anti-chick 488	703-546-155; Jackson Labs	1/1000
Donkey anti-goat 488	A11055: Molecular Probes	1/500
Donkey anti-rabbit 488	A21206; Molecular Probes	1/500
Donkey anti-rabbit 568	A10042: Molecular Probes	1/500
Donkey anti-rabbit biotinylated	ab7082; Abcam	1/100
Donkey anti-rat 488	A21208; Molecular Probes	1/500
Goat anti-mouse 488	A11029: Molecular Probes	1/500
Goat anti- sheep biotinylated	BA-6000; Vector Labs	1/100

### Tbr2 cKO Cell Comparison

Images of Nissl stained sections (40 μm thickness) were taken using a Leica light microscope (Leica DMR 500). Cells were counted in 100 μm widths using NeuroLucida (MBF Bioscience) and Adobe Photoshop CS6 (Adobe Systems Inc). Three sections each from *N* = 3 brains were analyzed for the cell counts and heterozygotes and homozygotes for the deletion were compared.

### Imaging and Image Analysis

Images were captured using a Zeiss LSM 710 confocal microscope (Carl Zeiss Microimaging). Similar image parameters (gain, pinhole, and wavelengths) were maintained for images from all brains. *Z*-stacks were taken individually for each channel and then collapsed to get maximum intensity projections. Images were analyzed using ImageJ (Image Analysis in Java, NIH) or Adobe Photoshop CS6 (Adobe Systems Inc.).

### Image Segmentation

Confocal images of multi-channel fluorescently labeled cells were analyzed using a histogram based multi-level thresholding algorithm (Arora et al. 2008) on MATLAB software (R2013a; Mathworks).

Briefly, this algorithm segments an image based on discreet threshold values, computed iteratively using the histogram. The optimum number of thresholds (*n*) required to segment the image (7–12 usually) is determined based on comparison of the PSNR (peak signal-to-noise ratio) values associated with different thresholding levels.

Finally, thresholded values were compared with the figure to verify that they showed similarly labeled cells.

We also used Imaris software (Bitplane) to analyze cell clones. For this, we used multichannel. lsm files to extract mean cell intensity in all 4 fluorescent channels. We then thresholded the intensity into 5 levels of 51 intensity points each. Based on this thresholding, each cell was awarded a cell code and reiteration of each code was checked in the dataset. Finally clonal sizes were rechecked for frequencies to understand distribution.

In all 3 brains labeled at E12.5 and perfused at P7 was used for this analysis, In each brain, 3 consecutive 150 µm slice was subjected to SeeDB as per published protocol (Ke et al. 2013) and imaged using confocal microscopy.

## Results

The expression of *Tbr2* has been previously described in the developing brain, and specifically in progenitors of the SVZ (Kimura et al. 1999; Englund et al. 2005). In contrast to other SVZ markers, such as the *Cux2* gene, Tbr2 is solely expressed in the IPCs while Cux2 expression can be seen in the upper cortical layers of the cerebral cortex and in some fate-restricted populations of radial glial cells (Zimmer et al. 2004), (Franco et al. 2012) thereby complicating its use as an IPC marker. Thus we chose to use a *Tbr2^Cre^* mouse line (Costello et al. 2011) to fate map the entire progeny of IPCs in the mouse brain.

### Analysis of IPC Derived Neurons in the *Tbr2^Cre^* Mouse

**Figure 1.**
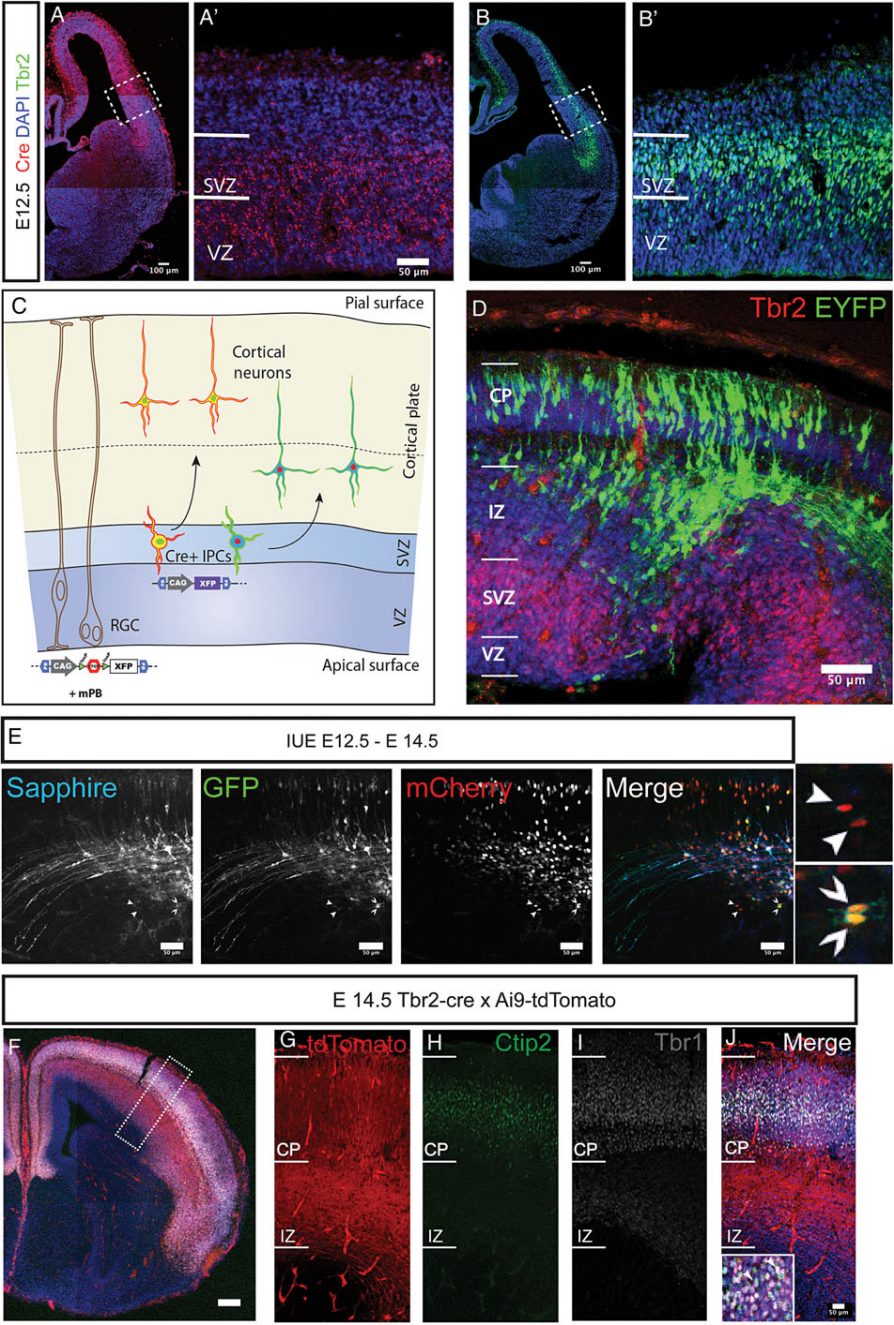
Analysis of IPC derived neurons in the *Tbr2^Cre^* mouse with stable transfection of fluoroproteins and Ai9-tdTomato reporter mouse. (*A,A*′) Expression of the cre-recombinase in *Tbr2*^*Cre*^ at E12.5 revealed with FISH. Cre mRNA is selectively expressed in the dorsal pallium and not in the subpallium in accordance with the known expression of Tbr2. Boxed region in *A* is shown with higher magnification in *A*′. (*B,B*′) Tbr2 immunoreactivity on an adjacent section to the one shown in *A* confirms the correlation between Tbr2 and Cre expression. Boxed region is shown with higher magnification in *B*′. (*C*) Schematic representation of the lineage analysis with stable transfection of CAG-STOP-floxed fluoroproteins using a constitutively expressing transposase plasmid by in utero electroporation. The constructs are taken up by the radial progenitors and passed on to IPCs where the recombination occurs leading to expression of fluorophores. (*D*) Single fluorophore (STOP-EYFP) transfection at E12.5 (collected at E14.5) revealed labeled cells in IZ, SP, and cortical plate, but not in VZ and SVZ, suggesting that the excision of the stop-floxed cassette occurs within the SVZ at the level of the Tbr2 positive IPCs (immunoreactivity for Tbr2 in red). (*E*) The method outlined in *C* can be extended to various CAG-STOP-floxed fluoroproteins to identify clonally derived cells. The shown example utilized 3 fluorophores (Sapphire, GFP, mCherry). Arrowheads indicate pairs of cells expressing the same combination of fluoroproteins. (*F*–*J*) Immunostaining sections from brains derived from breeding *Tbr2*^*Cre*^ and Ai9-tdTomato mouse lines at E14.5 revealed that Ctip2 (*H*) and Tbr1 (*I*) immunoreactive neurons are derived through Tbr2 positive IPCs. Scale bars: *A*,*B*,*F*: 100 μm, rest are 50 μm.

We first characterized the expression of the Cre-recombinase in the *Tbr2^Cre^* knock-in mouse by fluorescent FISH at E12.5. We found that the Cre-recombinase is selectively expressed in the cortical proliferative zones but not in the thin cortical plate (CP) at this stage (Fig. 1*A*,*A*′). We compared Cre mRNA expression with that of Tbr2 protein on an adjacent section and found it to be largely correlative in the SVZ (Fig. 1*B*,*B*′). Cells in the Tbr2 negative region of the VZ were also positive for Cre mRNA as shown previously indicating that transcription already occurs during the transition to IPC fate as with the *Tbr2* gene (Bulfone et al. 1999).

In order to label the progeny of IPCs, we employed 2 separate strategies. Firstly, we crossed the *Tbr2^Cre^* with the *Ai9-tdTomato* reporter mice (henceforth referred to as Ai9) to label all cells born from the Tbr2+ lineage across the brain during development. Secondly, we performed in-utero electroporation in *Tbr2^Cre^* pregnant mice at 12.5dpc (E12.5) in order to label lineages in a temporal fashion (*N* = 4 litters, *n* = 6 brains). To achieve this, we used a cocktail of transposase expressing plasmid and STOP-floxed fluoroprotein expressing plasmids. This cocktail labels clonal progenies of focally electroporated progenitors that pass through a Tbr2+ lineage with the same combination of fluorophores thus enabling their detection (Fig. 1*C*; (Garcia-Marques and Lopez-Mascaraque 2013; García-Moreno et al. 2014)).

We also performed short-term labeling (E12.5–E14.5, *N* = 2 litters, 4 brains) with only CAG-STOP-EYFP that marks cells exiting the SVZ (Fig. 1*D*). Importantly, no label was found in the ventricular zone that houses radial glial progenitors (Malatesta et al. 2000) displaying the specificity of the labeling. Immunostaining for Tbr2 showed only a small proportion of EYFP+ cells in the SVZ (Fig. 1*D*) with the majority in the intermediate zone (IZ) and CP. No colocalisation of EYFP+ cells with Tbr2 was observed indicating that expression of the fluorophores occurs during exit from SVZ.

In order to test for combinatorial and clonal fluorescent labeling of progenies, we electroporated a cocktail containing mT-Sapphire (membrane anchored), GFP (cytoplasmically targeted) and mCherry (nuclear targeted) at E12.5 and collected the brains 2 days later (*N* = 2 animals, 3 brains). As with the single fluorophore electroporation, most of the cells were positioned above the SVZ with some even sending axons through the IZ towards subcortical targets. We also observed 2 independent pairs of clones exiting the SVZ. The first pair (Fig. 1*E*; thick arrowheads) was negative for Sapphire, weakly positive for GFP and strongly expressing mCherry while the latter was positive for all 3 (thin arrowheads). Thus, clonal labeling of progenies of IPCs is possible using this method. In addition, the descent of axons from these cells (Fig. 1*E*,*F*) showed that a proportion of these cells are subcortically projecting and hence infragranular in nature. To confirm this, we performed immunostaining for Ctip2 and Tbr1 separately in E14.5 Ai9 brains (Fig. 1*F*–*J*). Both Ctip2 and Tbr1 colocalised with Ai9+ cells (Fig. 1*F*–*J*) above the IZ showing that infragranular layer neurons do arise from Tbr2+ IPCs during early neurogenic periods.

### Tbr2 Positive IPCs Derived Neurons Contribute to All Layers of the Cortex

**Figure 2.**
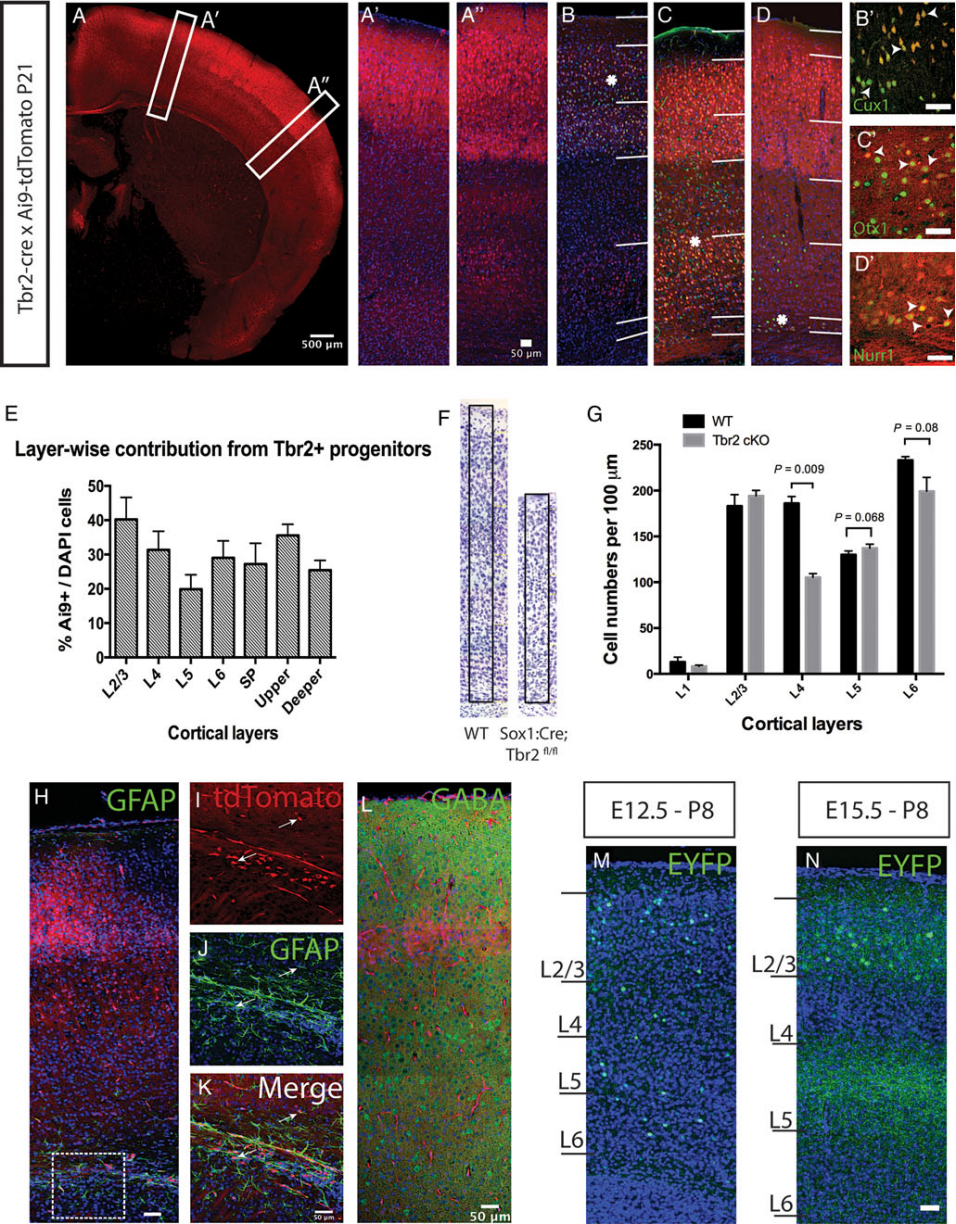
Tbr2-positive IPCs derived neurons contribute to all layers of the cortex. (*A*) Coronal section through a P21 brain derived from breeding *Tbr2^Cre^* and Ai9-tdTomato mouse. *A*′ and *A*″ were taken with higher power from regions indicated in *A*. (*B*–*D*) The cortical segment indicated in *A*″ was stained and imaged for Cux1 (*B*), Otx1 (*C*) and Nurr1 (*D*) immunoreactivity. Asterisks indicate the region in Layers 2–3 (*B*′), Layers 5–6 (*C*′) and SP (*D*′) where the high power images were taken. Arrowheads in *B*′–*D*′ indicate examples of double positive neurons. (*E*) The percentage of Ai9-tdTomato expressing cells from all DAPI stained cells in P21 sections (shown in *A*″) shows that 20–40% of all layers is derived through Tbr2+ IPCs in S1. Layers 2–3 shows the highest (40.2%) while Layer 5 is the least (19.8%). A total of 4 brains from 2 litters was used and at least 3 sections from each brain counted from. A minimum of 500 cells was counted in each section. (*F*) Images of Nissl stained somatosensory cortex from coronal sections of wild-type (WT) and Tbr2 conditional KO (*Sox1^Cre^*, *Tbr2^fl/f^*^l^) indicate reduced thickness. Arbitrary unit columns of 100 μm were boxed on the WT and Tbr2 cKO cortex. (*G*) Comparison of neuronal numbers in individual layers within a 100 μm column of wild type (WT) and Tbr2 conditional KO S1 cortex revealed significant reduction of Layers 4 and 6 and marginal increase in Layer 5. Three brains each from WT and cKO animals were used for cell counting where at least 3 sections from each brain at the S1 cortical level were counted. (*H*) GFAP immunohistochemistry on Ai9 brain sections at P7. Reactivity can be seen mostly in the white matter (boxed), but much less in other layers of the cortex. (*I*–*K*) Higher power images from the boxed region in *H* shows clusters of Ai9+ cells (*I*; arrows) in close proximity with GFAP+ cells (*J,K*; arrows) in the white matter of P7 brains. (*L*) Immunohistochemistry for GABA did not reveal any colocalisation with Ai9+ cells indicating that these were not GABA-ergic neurons. Three sections each from 3 brains were stained to check for colocalisation. (*M*,*N*) Single fluorophore (STOP-EYFP) labeling of clones born at E12.5 (*M*) and E15.5 (*N*). Early fate mapping at E12.5 (*M*) shows Tbr2+ IPCs give rise to cells across Layers 2–6 while at a later stage at E15.5 (*N*) cells can only be seen in the supragranular layers. Scale bars: a: 500 μm, rest are 50 μm.

As neurons continue to migrate well into postnatal stages, we addressed the final positions of neurons generated from all Tbr2+ IPCs in Ai9 brains at postnatal Day 21 (P21, *N* = 4 animals) after the completion of neuronal migration (Fig. 2*A*). We found Ai9+ cells across all layers of the cortex though this varied across different regions. Cingulate areas and the lateral cortex had Ai9+ neurons spread across the length of the cortex while in more dorsal regions fewer Ai9+ cells were present in infragranular layers (Fig. 2*A*,*A*″). We stained for known layer-specific markers such as Cux1, Otx1, and Nurr1 (Frantz et al. 1994; Nieto et al. 2004; Hoerder-Suabedissen et al. 2009) to confirm cell identities (Fig. 2*B*–*D*). Immunostaining for these markers revealed that Ai9+ neurons displayed fate identities in keeping with their positions. Majority of Ai9+ cells in supragranular layers also labeled for Cux1 while a smaller proportion of infragranular layers was positive for Otx1 (Fig. 2*B*′,*C*′, arrowheads). Surprisingly, a subset of Nurr1+ subplate (SP) cells was found to be Ai9+ (Fig. 2*D*′, arrowheads). SP neurons are among the earliest born cells in the cortex and are involved in mediating corticothalamic and thalamocortical innervation (McConnell et al. 1989; Hoerder-Suabedissen and Molnár 2013).

Additionally, we performed a quantitative analysis to ascertain the laminar contribution from Tbr2+ IPCs, in Ai9 brains at P21. We observed that Tbr2+ IPCs gave rise to neurons in all layers of the cortex (Fig. 2*E*) with varying proportions. Layers 2/3 and Layer 4 had the highest proportions of Ai9+ cells with 40.2% ± 6.4 (mean ± SD) and 31.3% ± 5.4, respectively, in the S1 somatosensory cortex. In comparison, Layer 6 and SP had fewer cells with 29% ± 4.9 and 27.2% ± 6. Layer 5 contained the fewest cells born through an IPC with 19.8% ± 4.2. Since Ai9+ cells were counted as percentage of all cells in the cortex (thereby including glial cells and interneurons in the denominator) the percentages of pyramidal glutamatergic neurons derived from Tbr2+ IPCs are under-represented in our observations. It is widely accepted that IPCs contribute towards upper-layer genesis (Tarabykin et al. 2001; Zimmer et al. 2004; Wu et al. 2005). However, our cell counting showed that while there was a significant difference between the contribution towards supragranular (35.55% ± 3.24) and infragranular layers (25.44% ± 2.84), the latter received a substantial contribution through IPCs. Looking at the cortical thickness as a whole, the contribution from IPCs was found to be 30.03% ± 1.95 in S1 showing that contribution towards cortical layers is more homogeneous than previously thought.

It was reported that *Tbr2* conditional knockout cortices showed a large decrease in the Cux1+ population of neurons with no significant decrease observed for Er81+ and FoxP2+ neurons (Arnold et al. 2008). However as these markers do not reveal all pyramidal cells in the respective layers, we undertook an unbiased layer-wise cell count (neurons and glia on Nissl stained sections) in the somatosensory cortex of *Sox1^Cre^; Tbr2^fl/fl^* conditional knockouts (Fig. 2*F*) at P8. Decrease in neuronal cell numbers was observed only for Layer 4 and Layer 6 of P8 cortex (Fig. 2*G*) with layer 4 showing a large decrease (∼43% of wild-type, *P* = 0.009, Multiple *t*-test with Sidak correction) and Layer 6 a relatively lesser reduction (14.5%, *P* = 0.08). None of the other layers showed an appreciable decrease except for a minor increase in the Layer 5 neuronal cell count (∼5%, *P* = 0.068). Hence the decrease in supragranular layers seems to stem from a decrease in Layer 4 only.

We stained for glial fibrillary acidic protein (GFAP) and gamma-amino butyric acid (GABA) in the P7 cortex to rule out glial and interneuron fate of Ai9+ cells (Fig. 2*H*–*L*). Expression of GFAP was largely limited to the white matter with some expression in Layers 1 and 5 (Fig. 2*H*). Some overlap of Ai9+ cells with GFAP+ processes (Fig. 2*I*–*K*) in the corpus callosum and rostral migratory stream could be seen which is in keeping with previous observations of Tbr2+ neuroblasts differentiating into glutamatergic interneurons in the olfactory bulb (Brill et al. 2009). On the other hand, GABA immunoreactivity did not overlap with Ai9+ cells in the cerebral cortex (Fig. 2*L*, a total of 12 sections were studied from 3 brains) maintaining that cells generated through the Tbr2+ lineage were glutamatergic as previously asserted (Hevner et al. 2006).

To further validate our observations, we also performed fate mapping by in utero electroporation of a Cre inducible fluorophore in *Tbr2^Cre^* cortex. For this, a mix of CAG-STOP-EYFP and mPB (transposase) plasmids was electroporated into the dorsal cortex at E12.5 and E15.5 followed by analysis at P8. When electroporation was performed at E12.5 (*N* = 3 brains each at P8), the resultant neurons generated were situated throughout the thickness of the cortex (Fig. 2*H*). Majority of cells were present in Layers 2–6, correlating with their birthdates being at the time of electroporation (Molyneaux et al. 2007) or afterwards. This also shows that the fluorophore cassettes were integrated in the radial glial progenitor genome. Cells were also observed in Layer 6 as expected (Fig. 2*M*) but fewer in number. In contrast pups collected from electroporations at E15.5 had labeled cells only in Layers 2–4 (Fig. 2*N*) and a strong band of fiber labeling could be observed in Layer 5; a known output target for Layer 2–3 neurons (Burkhalter 1989).

### Areal Variations in Cortical Numbers

As shown in Figure 2*A*, we observed consistent areal variations in Ai9+ cells in cortical layers throughout the cortex. This variation seemed to stem from differences in number of labeled cells in infragranular layers as dense labeling could be observed in the supragranular layers (Fig. 5*A*).

**Figure 3.**
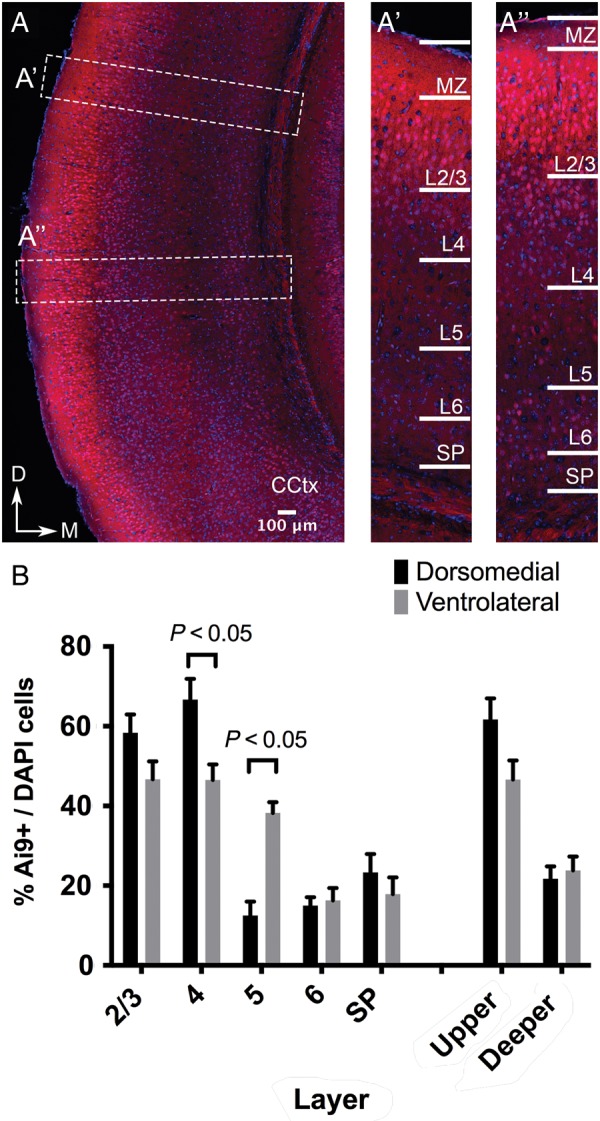
Areal variations in numbers of Tbr2 IP derived neurons with reciprocal differences between Layer 4 and 5 dorsomedial and ventrolateral cortices of the presumptive visual cortex. *A* The Tbr2 IPC derived Ai9+ cells are distributed across all cortical layers throughout the cortex. We compared their variation in number in selected areas labeled with boxes *A*′ and *A*″ in A (CCtx: Caudal Cortex). *A*′ and *A*″ We counted the number of Ai9+ cells in each layer between the dorsomedial (*A*′) and ventrolateral regions (*A*″). *B* Similar proportion of Ai9+ cells could be seen in Layers 2/3 (58.33 ± 4.6 vs. 46.67 ± 4.5, *n* = 2 brains, *P* = 0.12 multiple *t*-test with correction) but there was an appreciable difference in Layer 4 (66.67 ± 5.2 vs. 46.51 ± 3.9, *n* = 2, *P* = 0.048) and in Layer 5 (12.50 ± 3.5 vs. 38.24 ± 2.7, *n* = 2 brains, *P* = 0.014). No difference was noticed when all layers were grouped together suggesting against differences in cell densities (35.1 vs. 35.62). Scale bar 100 μm.

We decided to investigate this further at the level of the presumptive visual cortex (Fig. 3*A*) by counting the number of Ai9+ cells in each layer between the dorsal-medial (Fig. 3*A*′) and ventrolateral regions (Fig. 3*A*″).

In doing so, we found that while approximately equal Ai9+ cells could be seen in Layers 2/3 (58.33 ± 4.6 versus 46.67 ± 4.5, *n* = 2 brains, *P* = 0.12 Multiple *t*-test with correction), there was an appreciable difference in Layer 4 (66.67 ± 5.2 versus 46.51 ± 3.9, *n* = 2, *P* = 0.048) and in Layer 5 (12.50 ± 3.5 versus 38.24 ± 2.7, *n* = 2 brains, *P* = 0.014). No difference was noticed when all layers were grouped together suggesting against differences in cell densities (35.1 versus 35.62) (Fig. 3*B*).

These results suggest a difference in cell-cycle kinetics between these closely located regions also manifesting as reciprocal differences between Layers 4 and 5.

### Individual Clonal Analysis in the *Tbr2^Cre^* Mouse with Stable Transfection of Genetically Encoded Fluorophores Reveals Laminar and Areal Distribution

We were also interested in the clonal output from individual IPCs to the different cortical layers. For this we performed in utero electroporation as described above with the inclusion of plasmids carrying different fluorophores (mCherry, EYFP, EGFP and mSapphire; García-Moreno et al. 2014) targeted to different cellular locations (cytoplasmic, membrane, and nuclear) in combination with transposase at E12.5 and E15.5 and collected at P8 for analysis.

**Figure 4.**
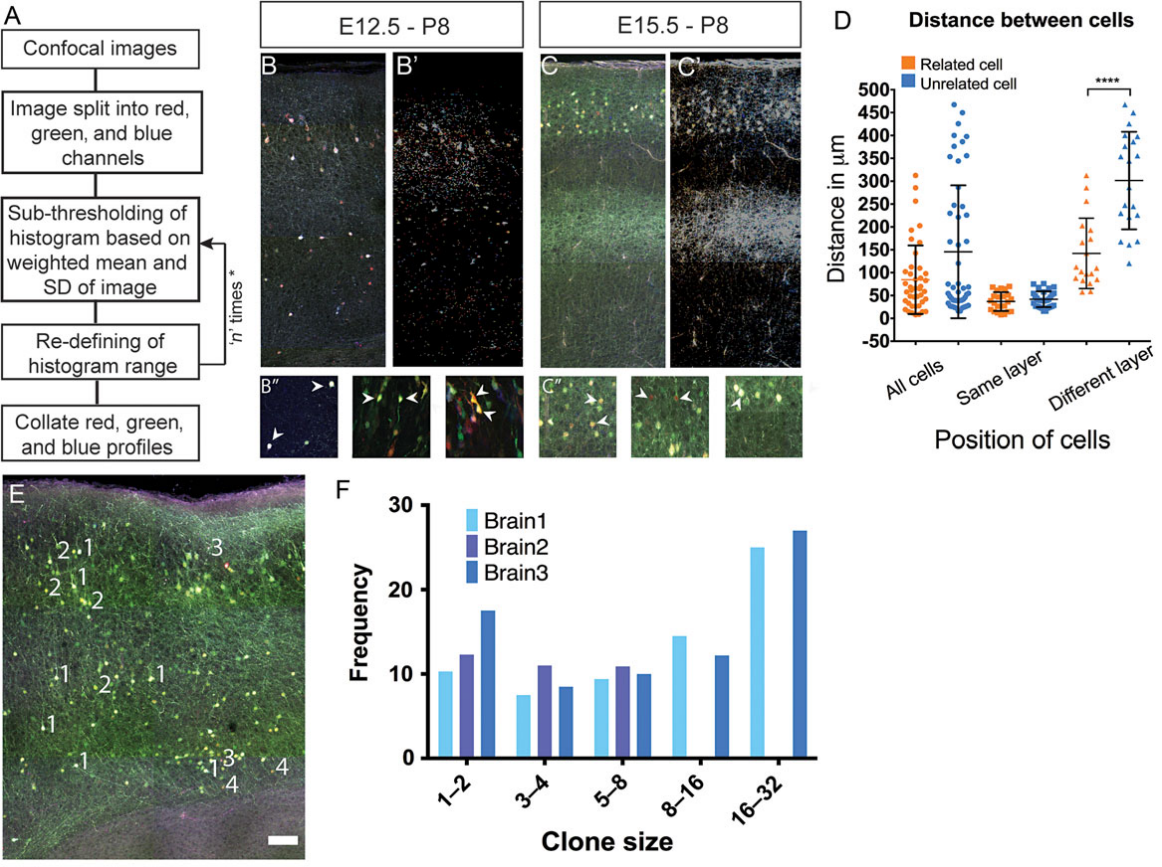
Analysis of the laminar and areal distribution of neurons derived from individual Tbr2+ intermediate progenitors in the *Tbr2^Cre^* mouse with stable transfection of fluoroproteins. (*A*) Algorithm for histogram based image clustering and segmentation. This algorithm was used to identify fluorescent cells that showed similar RGB properties and could be said to be clonal in origin. (*B*,*C*) Example of E12.5 (*B*) and E15.5 (*C*) electroporated brain labelled using CLoNe used for thresholding and the resulting thresholded image (*B*′,*C*′). Fluorescent cells of various hues can be seen in different layers. (*B*″,*C*″) Examples of clonally related cells identified using the algorithm. (*D*) Graph representing distance measured between clonally related and unrelated cells in same layer (36.8 ± 20.54 μm vs. 41.8 ± 17.30 μm), different layers (142.1 ± 76.8 μm vs. 294.9 ± 105.4 μm) and total (84.4 μm vs. 145.64 μm). Only cells clustered in different layers show significant difference as compared with unrelated cells (*P* < 0.0001, Mann–Whitney test). (*E*) Example of a 150 μm SeeDB clarified tissue section imaged under 4 channels and used for analysis. Numbers (in white) indicate clonal groups (1–4). Distribution of clonal group (1) is the most widespread radially, whereas 2 and 3 align in more radial fashion. Scale bar: 100 μm. (*F*) Graphical representation of clonal sizes and their frequency of occurrence. Clonal groups of 8–16 were found more frequently than those between 1 and 8. Also frequencies of larger clonal groups of 16–32 was the highest in the data analyzed from 3 brains electroporated at E12.5 and collected at P8. 3 serial sections each of 100–150 μm were studied and atleast 200 cells were studied in each section.

Clones were analyzed by histogram based thresholding algorithm (Fig. 4*A*) (Arora et al. 2008). Images generated above (Fig. 4*B*,*C*) were analyzed and distances between pixels holding similar values (representing clonally related cells) measured. We found that related cells in the same layer were separated by a mean distance of 36.8 ± 20.5 μm (*n* = 23 cell pairs across 4 brains) while those in different layers had a separation of 142.1 ± 76.8 μm (*n* = 19 cell pairs across 3 brains) (Fig. 4*D*). Additionally, clonally related cells separated by cortical layers were clustered closer than unrelated ones (142.1 ± 76.8 μm vs. 294.9 ± 105.4 μm; *P* < 0.0001) (Fig. 4*D*). However, this did not apply to clonal clusters within the same layer or to the sum of the 2. As clonally born neurons are known to disperse long distances (Walsh and Cepko 1993), we decided to use tissue clearing methods such as SeeDB (Ke et al. 2013) to image and analyze labeled neurons in serial section each of 100–150 μm thickness. Our analysis on brains electroporated at E12.5 revealed that depending on the number of cells labeled (between 200 and 400 per brain), the distinct labeling patterns generated varied between 20 and 30 (data not shown). We observed that the larger clone sizes had cells distributed between more than one radial column suggesting that IPCs can give rise to neurons in adjacent radial columns (Fig. 4*E*).

We also saw that clone sizes of >8 were generated more often that those between 1 and 8 (Fig. 4*F*) by almost a factor of 2 (19.7 vs. 10.8, data from 3 different brains at P7 and 200–400 cells per brain, *t*-test with Sidak correction). Further, frequencies of clonal size of >16 showed the highest representation compared with other sizes. As these clonal sizes can be achieved only when both early and late born IPCs are labeled, this further demonstrates the fidelity of our method. Additionally, it also shows that more IPCs are born during early cortical development (thus giving larger clonal sizes). We also found that not all combinations of an arithmetic progression were represented in the clonal distribution and most values between 9 and 16 (except for 11, 12, and 16) were absent in our analysis.

### Identity of Tbr2 Derived SP Neurons

SP cells have been described as a transient neuronal population that undergoes programmed cell death during late embryonic and early postnatal development (Price et al. 1997). However, a significant proportion of these cells do survive and are labeled by a variety of immunohistochemical markers (Kostovic and Rakic 1980; Del Río et al. 2000; Okhotin and Kalinichenko 2003; Hoerder-Suabedissen and Molnár 2013). Some studies have postulated that the acetylcholine esterase expressing interstitial white matter cells might be the surviving vestiges of the SP neurons (Kostovic and Rakic 1980; Chun and Shatz 1989).

**Figure 5.**
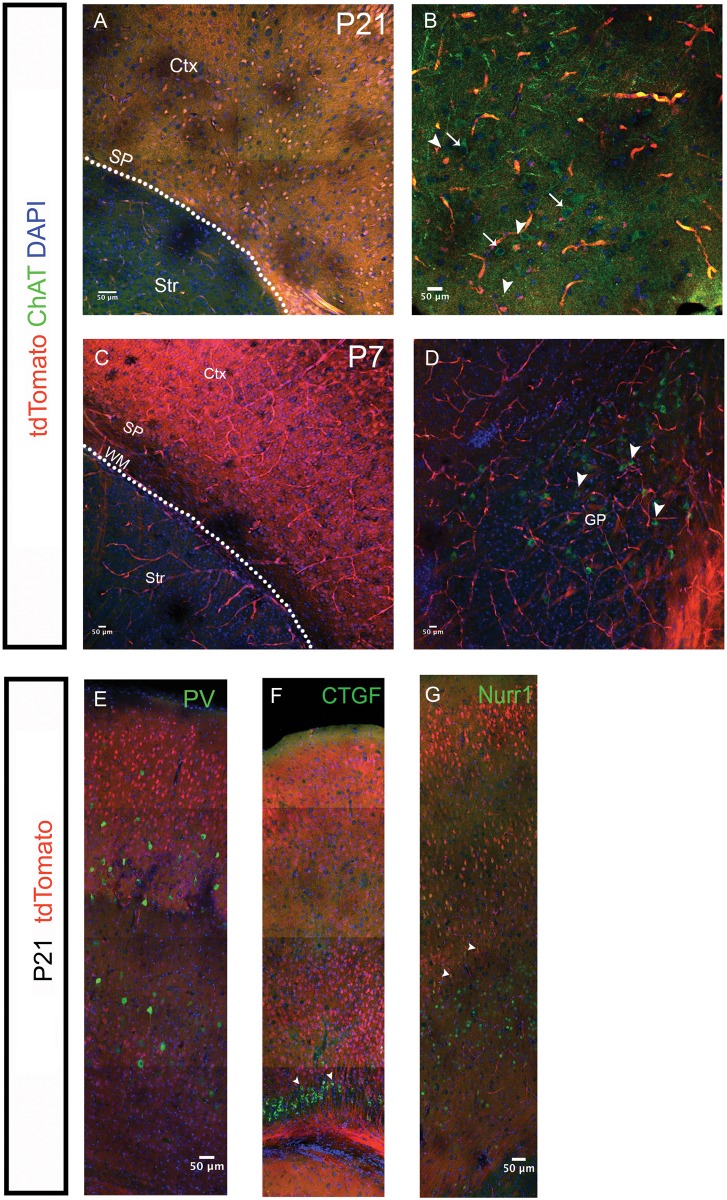
Characterisation of Tbr2+ IPC derived SP neurons for ChAT expression and various markers at P7 and P21. (*A*–*D*) Tbr2 derived SP cells (tdTomato) were stained from ChAT at P21 (*A*,*B*) and at P7 (*C*,*D*). No ChAT+ tdTomato+ cells in the SP or white matter at P21 or P7 were found (*n* = 3 brains) despite ChAT+ cells being present in the globus pallidus (arrows in *B*). Similar to P21, no ChAT+ cells were seen at this age in SP/WM region either despite their presence in the ventrobasal areas of the forebrain (*D*). (*E*–*G*) Tbr2 derived SP cells were positive for Cplx3 and Nurr1 (arrows in *F*,*G*) but not for the interneuron marker parvalbumin (*E*). SP, subplate; Str, striatum, WM, white matter; Ctx, cortex; GP, globus pallidus. Scale bars 50 μm.

To examine if Tbr2 derived SP cells remain as interstitial cells, we chose to look for AChE+ tdTomato+ cells in the SP and white matter at P21 and P7 by using an antibody against ChAT. However, at P21, we could find no ChAT+ cells in the SP/WM region (*n* = 3 brains) despite ChAT+ cells being present in the globus pallidus (Fig. 5*A*,*B*). In order to see whether ChAT+ cells are present during early postnatal ages, we also looked at P7 cortices. Similar to P21, no ChAT+ cells were seen at this age either despite their presence in the ventrobasal areas of the forebrain (Fig. 5*C*,*D*).

As the SP has been described to be a heterogeneous population (Del Río et al. 2000; Hoerder-Suabedissen et al. 2009; Hoerder-Suabedissen and Molnár 2013), we performed immunostaining for other SP markers. We found that Tbr2 derived SP cells were not positive for the interneuron marker parvalbumin (Fig. 5*E*) but expressed other SP markers such as CTGF and Nurr1 (Fig. 5*F*,*G*).

### Extracortical Contributions of Tbr2+ Cells

**Figure 6.**
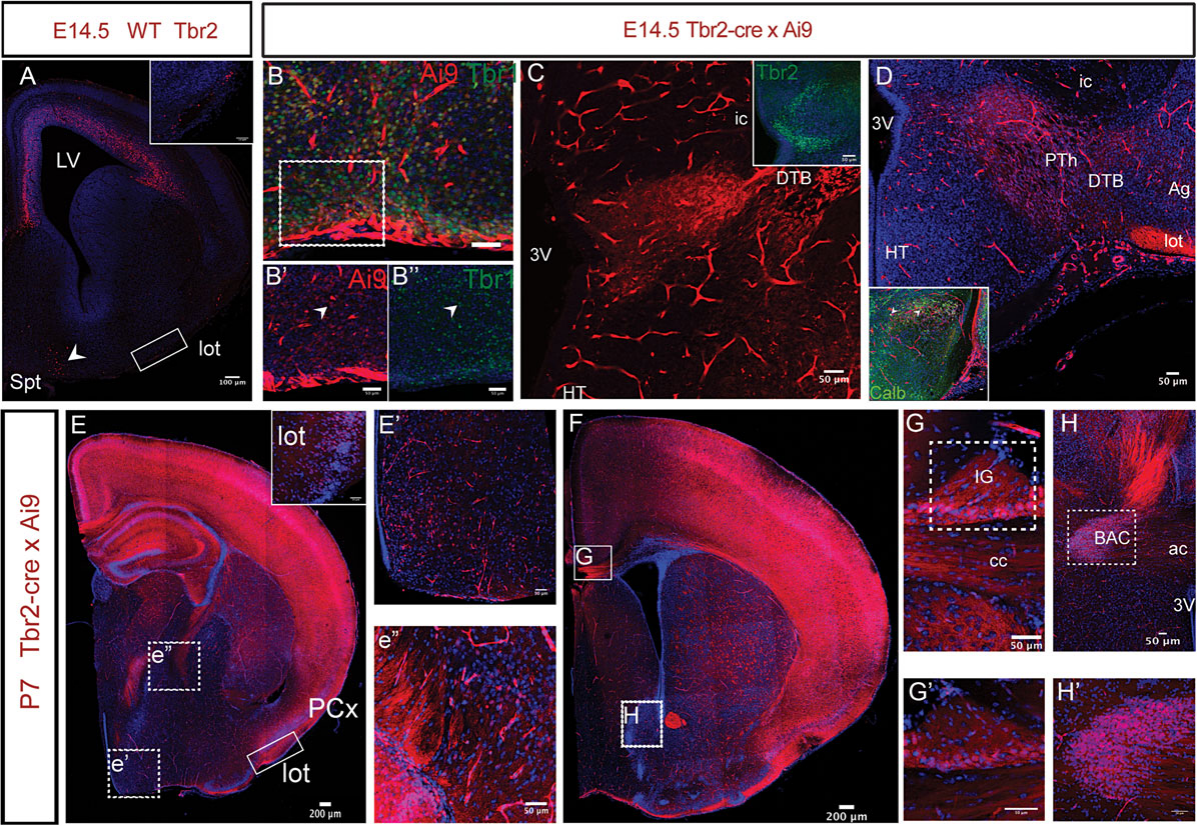
Extracortical contributions of Tbr2+ IPCs. (*A*) Tbr2 immunoreactivity is detected at E14.5 in the septum (Spt, arrowhead) and in the lateral olfactory tract (lot). Inset shows expression in the lot from boxed area. LV, lateral ventricle. (*B*) Ai9+ (red) cells in the septal regions colocalise with Tbr1 (green) in the *Tbr2^Cre^* bred with Ai9-tdTomato. Tbr1 expression (*B*″, arrowhead) overlaps with Ai9 (*B*′, arrowhead) indicating glutamatergic nature of these cells. *B*′ and *B*″ are taken from the boxed region of an adjacent section. (*C*) Tbr2 is also expressed in the prethalamus (PTh) as indicated by Ai9+ cells at E14.5. Cells can be seen on either sides of the DTB. Inset shows expression of Tbr2 in the thalamic eminence. (*D*) On a more posterior section Ai9+ cells can be seen crossing the DTB and also in the lot at that level. Inset shows that some cells in the prethalamus coexpress calbindin (arrowheads). Abbreviations: 3V: 3rd Ventricle, HT: Hypothalamus, ic: internal capsule, Ag: amygdala, lot: lateral olfactory tract. (*E*) Coronal section from a brain derived from the breeding of *Ai9-tdTomato* and *Tbr2^Cre^* at P7 shows expression is maintained in the lot. Inset shows labeled cells in higher magnification in lot. PCx: piriform cortex. *E*′ and *E*″ show that cells in the hypothalamus and prethalamus also continue to express Ai9 at postnatal ages (taken from *E* at the sites of *e*′ and *e*″). (*F*) Cell of the indusium grisuem (IG; *G*,*G*′) and bed nucleus of anterior commissure (BAC; *H*,*H*′) express Ai9 in a P7 *Tbr2^Cre^* × *Ai9* section. (*G*,*H*) show expression in a corresponding section. (*G*) Expression of Ai9 can be seen in the cells of the IG next to the midline and above the fibers of corpus callosum (also labeled by Ai9). Inset shows higher magnification. (*H*) Ai9 expression can be seen in BAC as well as in the anterior commissure. Inset shows higher magnification from the boxed region. Scale bars: a: 100 μm, EF: 200 μm while rest is 50 μm.

In addition to our fate mapping in the cortex, we also observed Tbr2 expression in previously undescribed regions. We observed Tbr2 immunoreactive cells in the embryonic septum and lateral olfactory tract (lot) (Fig. 6*A*) as well as in a small population of migratory-shaped cells along the margins of the rostral medial telencephalic wall proceeding ventrally towards the septum. A similar migration has been previously described (García-Moreno et al. 2008; Ceci et al. 2012) though the nature of the progenitors was not commented on. We also observed a large number of cells in the ventro-medial cortex and septum at E14.5 in coronal sections of Ai9 brains (Fig. 6*B*) a large proportion of which colocalised with Tbr1 (Fig. 6*B*′,*B*″; arrowhead) indicating that they are glutamatergic in nature. Ai9+ cells in the lot were also positive for calbindin (Fig. 6D inset) as reported previously (García-Moreno et al. 2008; Ceci et al. 2012).

Ai9 labeled cells were also observed at thalamic eminence and the prethalamus at E14.5 and appeared to cross the diencephalon–telencephalon boundary (DTB) seemingly emanating from the ventricular zone of the third ventricle (Fig. 6*C*,*D*). It has been reported that cells from this region migrate and contribute towards the posterior accessory olfactory bulb (pAOB) (Huilgol et al. 2013) and the medial amygdaloid nucleus (García-Moreno et al. 2010). Thus, it seems that Tbr2+ cells contribute towards the development of the vomeronasal system both by generating neurons that set up the lot and also by contributing towards the stream of neurons migrating from the prethalamus towards the pAOB.

We also examined sections from postnatal Ai9 brains (P7). As expected, we found labeled cells in the lot and the olfactory tubercle (Fig. 6*E*, inset).

Our analysis of labeled cells also revealed Tbr2+ derived cells in other regions. We observed expression of Ai9 in the hypothalamus and prethalamus (Fig. 6*E*′,*E*″). Ai9+ cells in the hypothalamus however failed to colocalise with either tyrosine hydroxylase (TH) or choline acetyltransferase (ChAT; data not shown) indicating that they were generated from a different progenitor pool and were not neurons of the basal forebrain. In addition, Ai9 staining could also be seen in the cells of indusium griseum, bed nucleus of anterior commissure (BAC) and the anterior commissure itself (Fig. 6*F*–*H*).

## Discussion

Our results show that in contrast to accepted paradigms, Tbr2+ IPCs contribute towards all layers of the cortex including the earliest born SP layer. As IPCs are an acquired progenitor group during cortical evolution, it suggests that the expansion of the cortex has been more homogeneous among all layers than previously thought (Tarabykin et al. 2001). These observations are further supported from our current quantitative analysis of the somatosensory cortex of *Sox1^Cre^; Tbr2^fl/fl^* conditional knockouts where we describe reduction in cell numbers in all layers. Our study provides the first analysis on the spatial relation between clonally derived neurons from IPCs and clonal size. Accordingly, clonally related cells positioned in different layers cluster closer than unrelated cells. Finally, our study identified extracortical contributions of Tbr2+ expressing progenitors that are involved in the generation of glutamatergic neurons of the lateral olfactory tract, the olfactory tubercle, and ventromedial hypothalamic nucleus.

The contribution from Tbr2+ progenitors towards the cerebral cortical layers has not been studied directly, quantitatively and systematically. While its origin has been well described (Haubensak et al. 2004; Miyata et al. 2004; Noctor et al. 2004), the fate of its progenies has not been followed into later postnatal stages. Ambiguity persists regarding the contribution towards the upper and lower layers. A study utilizing *Tbr2* conditional knockout (Arnold et al. 2008) describes a decrease of immunoreactivity for an upper layer marker while another report (Sessa et al. 2008) describes a loss in both upper and lower layers. In the latter case however, haploinsufficiency at the *Foxg1* locus is known to decrease the specification of IPCs by ∼38% (Siegenthaler et al. 2008) and is associated with a decrease in supragranular layer neurons (Eagleson et al. 2007; Siegenthaler et al. 2008). Thus use of this Cre line would lead to erroneous interpretation. Using *Tbr2::GFP*, it has been reported that early born IPCs contribute towards infragranular layers (Kowalczyk et al. 2009). However, this is an indicative assumption as the GFP label is impermanent and laminar fates of neurons cannot be determined using this transgenic.

In contrast, transgenics with a single copy of *Tbr2* develop normally and do not show reduced expression (Arnold et al. 2008, 2009). Thus the *Tbr2^Cre^* mouse line is better suited for fate-mapping of IPCs as shown by us using immunohistochemistry and reporter mouse lines. Using this line, we show that Tbr2+ IPCs give rise to both upper and lower layer neurons but in different proportions. Importantly, we describe that IPCs give rise to a subpopulation of SP neurons proving important during early corticogenesis and circuit formation.

Our study also demonstrated that Tbr2+ IPCs do not produce GABAergic or astrocytic cells. It is reported that the rodent somatosensory cortex contains ∼36% glia and ∼64% neurons (Ren et al. 1992). Further, among neurons, ∼25% is constituted by GABAergic interneurons with excitatory cells making the majority of cells (Ren et al. 1992). In simpler terms, for every 100 cells counted in the somatosensory cortex of rodents, 64 cells are neurons of which 16 cells would be interneurons. Considering from results shown above that Tbr2+ IPC derived neurons account for 30% of all cortical cells, it implies that ∼62.5% of excitatory neurons (30/48) are produced via Tbr2+ IPCs with the number being even higher in upper layers and remaining constant for lower layers (exact calculations are difficult since astrocytes and oligodendrocytes show layer specific clustering). This number differs from the predictive studies that use pH3+ cell numbers in the SVZ to calculate the contribution from IPCs (Martínez-Cerdeño et al. 2006) perhaps due to difference in the region used for analysis. Our study hence does not support the idea for an exclusive path for the generation of glutamergic neurons but instead suggests most glutamatergic neurons to be born via a RGC to IPC to neuron pathway.

Our layer-wise cell counts from the conditional knockout as well as from the Ai9 brains indicates that while neurons born from IPCs come to lie across all cortical layers, the decrease in the conditional knockout is only seen in Layers 4 and 6. This suggests redundancy in *Tbr2* function observed in relation to other layers (than L4) during cortical neurogenesis (Arnold et al. 2008). Single cell profiling has described gene clusters that are expressed in the SVZ and might be involved in specifying neuronal fate (Kawaguchi et al. 2008). In addition, *Tbx1*, another T-box gene that has been described to affect proliferation of IPCs is correlated with DiGeorge Syndrome (Meechan et al. 2009) leading to a decrease in supragranular layer neurons. Such clusters and paralogs might rescue some of the deleterious effects of Tbr2 gene knockout. This problem might be magnified in animals with larger brains having a larger Tbr2+ population.

Analysis of clonally generated cells from Tbr2+ IPCs shows that they do not disperse long distances but are in close proximity with one another. Also, we show that clonal clusters are located within limits that suggest their arrangements in columns as previously described (Mountcastle 1997). We also show for the first time that IPCs can generate neurons towards adjacent radial columns despite coming from the same RGC pool. It will be interesting to further study whether the clonally related cells in our study are also functionally related (Yu et al. 2009; Li et al. 2012).

The regulation of cortical patterning and arealisation is attributed to multiple factors. Studies have shown an inherent difference in cell-cycle kinetics between different regions at embryonic ages that lead to differences in cortical numbers (Dehay et al. 1993; Polleux et al. 1997; Dehay and Kennedy 2007). This has further been supported by artificial modification of cell-cycle parameters leading to changes in cortical numbers (Lukaszewicz et al. 2005; Pilaz et al. 2009) and also by genetic and transcriptomic studies (Bishop 2000; Pinto et al. 2009; Elsen et al. 2013). In addition, modulation of thalamic input has also been shown to influence cortical parcellation (Dehay et al. 1996). However, the contribution of specific progenitor cells in areal cortical number differences has not been looked into in detail.

Using the fate mapping approach, we show a difference in cortical numbers in specific laminae between dorsomedial and adjoining ventrolateral cortex at the level of both somatosensory and visual cortices indicating that differential IPC division between adjoining areas might lead to areal differences in the mature cortex.

This study also describes expression of *Tbr2* in regions outside of the cerebral cortex namely in the prethalamus, embryonic septum, and hypothalamus. The septal expression is particularly interesting as it aides in the development of the lot (García-Moreno et al. 2008; Ceci et al. 2012), which has been shown to be affected in the conditional *Tbr2* knockout (Arnold et al. 2008) thus arguing for a role of these cells in extracortical regions. We also show expression in the prethalamus during embryonic development. A case can hence be made for the involvement of Tbr2+ IPCs in specification and development of these regions and ultimately to the development of the brain. However, it remains to be seen as to whether the transcriptional regulation in these regions is similar to that studied in the SVZ (Sessa et al. 2008; Cameron et al. 2012; Elsen et al. 2013).

Finally, we show expression in the bed nucleus of anterior commissure and the indusium griseum, regions that are closely located with major fiber tracts of the brain, i.e., the anterior commissure and corpus callosum both of which are absent in the conditional *Tbr2* knockout mouse (Arnold et al. 2008) and in human with mutations in the TBR2 gene (Baala et al. 2007).

Intermediate progenitors have been hypothesized to be an important addition in the evolutionary expansion of the cerebral cortex (Martínez-Cerdeño et al. 2006; Cheung et al. 2007, 2010). It is also known that a mutation spanning the TBR2 locus in humans causes microcephaly with polymicrogyria (Baala et al. 2007). However, from mouse models of loss of function of *Tbr2* it is not clear as to which populations of cortical neurons are affected. In this study, we have shown the pan-cortical contribution from IPCs and to several other regions crucial for the development of the rodent brain. The evolutionary impact of IPCs will thus have to be revisited to study if the expansion is limited to the neocortex or if it applies to these other regions of expression as well. At a clonal level, it would again be interesting to compare differences among mammals with an expanded germinal zone (Fietz et al. 2010; Reillo et al. 2010; García-Moreno et al. 2012; Kelava et al. 2012) as well as with avian and reptilian counterparts. However, this might need designing of new experimental strategies.

## Authors’ Contributions

The study was designed by N.A.V., F.G.-M., and Z.M. N.A.V. performed the fate-mapping and clonal analysis experiments. F.G.-M. performed some of the clonal analysis experiments and established the method used for Figure 3. S.A. wrote the algorithm for image segmentation. A.C. performed and analyzed the Tbr2cKO cell counting. *Tbr2^Cre^* mouse strains and Tbr2-deficient tissues were generated and provided by S.J.A. and E.J.R. N.A.V., F.G.-M., and Z.M. wrote the paper.

## Funding

This study was funded by Medical Research Council, UK and Biotechnology and Biological Sciences Research Council, UK grants to Z.M. S.J.A. was supported by the German Research Foundation (DFG; Emmy Noether Programme AR 732/1-1). F.G.-M. is funded by Human Frontiers Science Program and N.A.V. is funded by Goodger and Felix Scholarships. Funding to pay the Open Access publication charges for this article was provided by University of Oxford RCUK Access Block Grant.
